# Incompetence of Vector Capacity of *Rhipicephalus bursa* to Transmit *Babesia aktasi* following Feeding on Clinically Infected Goat with High Level of Parasitemia

**DOI:** 10.3390/vetsci11070309

**Published:** 2024-07-10

**Authors:** Mehmet Can Ulucesme, Sezayi Ozubek, Munir Aktas

**Affiliations:** Department of Parasitology, Faculty of Veterinary Medicine, University of Fırat, 23200 Elazığ, Türkiye; sozubek@firat.edu.tr (S.O.); maktas@firat.edu.tr (M.A.)

**Keywords:** *Babesia aktasi*, experimental study, *Rhipicephalus bursa*, transstadial and transovarial transmissions, vector competence

## Abstract

**Simple Summary:**

The tick species involved in the transmission of *Babesia aktasi*, which is widespread in the Mediterranean region, is unknown. However, studies have shown that *Rhipicephalus bursa* is the most common tick species in the regions where the prevalence of *B. aktasi* is widespread. This finding increases the possibility that *R. bursa* may serve as a vector for the transmission of *B. aktasi* and encourages the evaluation of the vector competence of *R. bursa*. For this purpose, clinical babesiosis was induced in an immune-suppressed indigenous donor goat. *Babesia* spp.-free *R. bursa* larvae (*n* = 2000) and adults (*n* = 25) obtained from laboratory colonies fed on the donor goat. Following oviposition, PCR analysis of engorged female carcasses and engorged nymphs revealed the presence of *B. aktasi*, whereas no positivity was found in unfed larvae and adult ticks. The subsequent developmental stages of these ticks were used to infest three additional immune-suppressed goats. No clinical signs of babesiosis were observed in the infested goats. Moreover, molecular analysis did not detect DNA in the goats. These results demonstrated that *R. bursa* does not transmit *B. aktasi*, neither transovarially nor transstadially.

**Abstract:**

A recent molecular survey revealed a high prevalence of *Babesia aktasi* in indigenous goats from the Mediterranean region of Türkiye, coinciding with heavy *Rhipicephalus bursa* infestations. This geographical overlap has raised the possibility that *R. bursa* may serve as a vector for the parasite. To evaluate the potential of *R. bursa* to serve as a vector for the parasite, an experimental study was conducted in indigenous goats. An immune-suppressed donor goat was intravenously injected with 15 mL of the cryopreserved *B. aktasi* stabilate, resulting in severe clinical babesiosis and parasitemia. Subsequently, *R. bursa* larvae and adults derived from *Babesia*-free laboratory colonies were allowed to feed on the infected donor goat. After oviposition, engorged female carcasses, representative engorged nymphs, unfed larvae, and adult pools were used for DNA extraction and PCR analysis. No PCR positivity was detected in any of the DNA samples, except for those with engorged female carcasses and nymphs. Three immune-suppressed recipient goats were infested with the unfed immature and mature ticks consuming the blood of a donor infected with *B. aktasi*. No clinical or parasitological findings were encountered in the recipient for 40 days post-infestation. These findings indicated that *R. bursa* was not a competent vector for *B. aktasi*.

## 1. Introduction

Babesiosis is a protozoan disease caused by intra-erythrocytic parasites belonging to the phylum Apicomplexa and genus *Babesia* [1,2]. The disease, characterized by clinical signs such as high fever, anemia, jaundice, hemoglobinuria, and death in hosts, is a common tick-borne infection in mammals [3,4,5,6]. The life cycle of *Babesia* species occurs between vertebrate hosts and tick vectors [7,8]. Shortly after an infected tick attaches to its host to feed on its blood, it injects *Babesia* sporozoites into the host along with its saliva. *Babesia* species belonging to the *Babesia* sensu stricto (s.s) clade (clade VI) are transmitted by ixodid tick vectors through both transstadial and transovarial transmissions [8,9]. Numerous ixodid tick species have been identified as vectors of *Babesia* species [1,10]. *Babesia* species or genotypes, such as *B. ovis, B. motasi*, *Babesia* sp. Xinjiang, and *Babesia* sp. BQ1 (Lintan), which cause babesiosis in sheep and goats, are transmitted by the *Rhipicephalus*, *Haemaphysalis*, and *Hyalomma* genera [1,11,12,13,14,15]. It has been noted that *B. ovis*, the main etiological agent of clinical babesiosis in sheep and goats, can be transmitted by *Rhipicephalus bursa*, *R. sanguineus*, *R. turanicus*, *R. evertsi*, and *Hyalomma excavatum* [16,17,18,19]. However, it has been found that transovarial transmission occurs exclusively by *R. bursa*, which feeds on sheep and goats during both immature (larval and nymphal ticks) and adult stages [13,15]. *Babesia motasi* is transmitted by *Haemaphysalis punctata*, while the vector for *B. crassa* remains unknown [10].

Advancements in molecular biology have enabled the development of tools that facilitate rapid and precise diagnosis of babesiosis. In parallel with the development of diagnostic techniques, several novel ovine *Babesia* species or genotypes, such as *Babesia* sp. Xinjiang and *B. motasi*-like, have been added to the list [2,20,21]. Recently, a novel *Babesia* species infecting indigenous goats was detected in the Mediterranean region of Türkiye [22] and then isolated from a naturally infected goat and named *Babesia aktasi* [23]. Our molecular survey carried out in the same region indicated that *B. aktasi* infections were highly prevalent in the goat populations, with a prevalence of 22.5% (113/503) [24]. Then, an experimental study revealed that *B. aktasi* caused typical clinical findings of babesiosis (fever, anemia, icterus, and hemoglobinuria) resulting in death in immune-suppressed indigenous goats [25]. The development of effective control strategies against tick-borne diseases lies in identifying the tick vector transmitting the pathogen [9]. The tick vector transmitting *B. aktasi* has not yet been identified. The tick survey demonstrated that the most common tick infesting goats in the region where the prevalence of *B. aktasi* is widespread was the two-host tick *R. bursa* [24]. This epidemiological overlap indicates that the potential tick vector responsible for the parasite transmission could be *R. bursa*. However, there is a need for an experimental vector competence study to confirm this assumption. Therefore, we focused on evaluating the vector competence of *R. bursa* for the transstadial and transovarial transmissions of *B. aktasi*.

## 2. Materials and Methods

### 2.1. Ticks and Parasite

The two-host ticks (*Rhipicephalus bursa*) used in this study were obtained from *Babesia* spp.-free colonies maintained at the Department of Parasitology, Veterinary Faculty of Fırat University, Elazığ, Türkiye. Briefly, adult *R. bursa* ticks were collected from naturally infested healthy domestic ruminants in a *B. aktasi*-free area, Elazığ Province in eastern Türkiye. Among them, 12 engorged female ticks collected from cattle were incubated at 25 ± 1 °C, 70 ± 10% relative humidity (RH). After oviposition, the engorged female carcasses and egg pools containing 100 eggs from the egg mass laid by each of 12 female ticks were screened by nested PCR (nPCR) for pathogen detection (*Babesia* spp., *Theileria* spp., *Anaplasma* spp., and *Ehrlichia* spp.) [23]. According to these results, 4 new-generation larval batches (F1) that were nPCR-negative for the detected pathogens’ DNA were chosen and used to establish a laboratory colony. F1 larval ticks were allowed to feed on a New Zealand White rabbit and engorged nymphs were obtained. After molting to the next stage, the unfed adult ticks were fed on a *Babesia* spp.-free immune-suppressed indigenous goat. After confirming that the ticks were free of *Babesia* species, the colony was started in our tick facility at Fırat University, Veterinary Faculty, Department of Parasitology. The ticks utilized in this experimental study were the F2 and F3 generations of the colony.

The *B. aktasi* isolate used in this study was obtained from a naturally infected indigenous goat and then passaged in another splenectomized goat. Additionally, the *B. aktasi*-positive animal was also screened for the presence of other tick-borne pathogens, such as *Anaplasma* spp. and *Theileria* spp., to ensure that no co-infections were present. Blood samples were collected from the passaged goat at the peak parasitemia and cryopreserved in liquid nitrogen as a blood stabilate containing 8% dimethyl sulfoxide [23]. All experimental procedures were approved by the Fırat University Animal Experiments Local Ethics Committee (session number: 2021/12).

### 2.2. Selection of Experimental Goats

**Figure 1:**
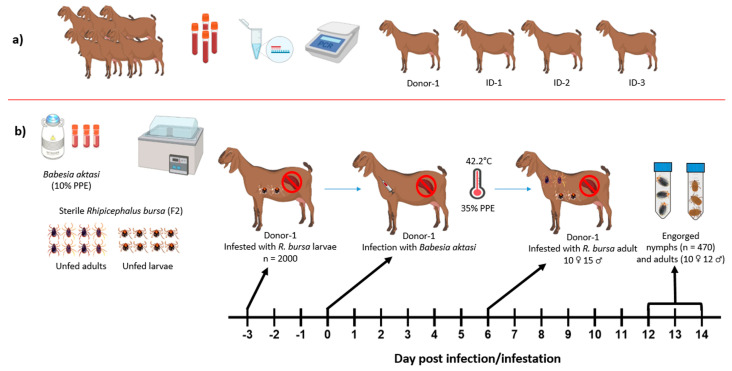
Representative schema of experimental study design. (**a**) Determination of pathogen-free (*Babesia*, *Theileria*, and *Anaplasma*) indigenous goats; (**b**) infection of the immune-suppressed indigenous donor goat with *B. aktasi* stabilate (PPE 10%) and infestations by immature and adult stages of *R. bursa*. Figure 1 was created using BioRender.com (https://www.biorender.com/ accessed on 12 June 2024). (PPE: percentage of parasitized erythrocytes).

*Babesia*-, *Anaplasma*-, and *Theileria*-free indigenous goats were used in this study. For this purpose, EDTA blood samples were collected from apparently healthy 7-month-old indigenous male goats from a breeding farm located in a village of Elazığ Province, Türkiye. Peripheral thin blood smears from the ear vein of the same goats were prepared for microscopic examination. The samples were brought to the laboratory (Fırat University, Faculty of Veterinary Medicine, Department of Parasitology). Thin blood smears and DNA isolated from EDTA blood samples were tested to ensure the absence of the selected pathogens screened by microscopy and nPCR for the presence of *Babesia*, *Theileria*, and *Anaplasma* species [24,26]. Four goats (#Donor-1, #ID-1, #ID-2, and #ID-3) determined to be negative were purchased and transported to Fırat University Animal Hospital for splenectomy (Figure 1a).

### 2.3. Splenectomy and Post-Operative Care

The goats underwent splenectomy using standard anesthesia, analgesic, and surgical techniques at the hospital [25,27]. Following splenectomy, they were transported to the small ruminant unit where the experiment would take place (Ministry of Agriculture, Elazığ Veterinary Control Institute, Elazığ, Türkiye). Each of the goats was kept in a separate tick-free pen at the unit. As post-operative care, the goats were stabilized by administering intravenous 0.9% physiological saline (250 mL) and 5% dextrose (250 mL) once a day for 2 days. In order to prevent possible bacterial infections, a 5-day course of intramuscular antibiotic treatment was administered after splenectomy. Additionally, antibiotic spray (Neo Caf, Oxytetracycline, aerosol spray suspension, 200 mL, MSD, Rahway, NJ, USA) was applied to the surgical wound every day for a week. The goats were fed with dry clover and crushed barley, and water ad libitum. Three weeks after the splenectomy, microscopy and nPCR were repeated, and it was confirmed that the goats were free of *Babesia*, *Theileria*, and *Anaplasma* species.

### 2.4. Transmission Experiments of Babesia aktasi

**Figure 2:**
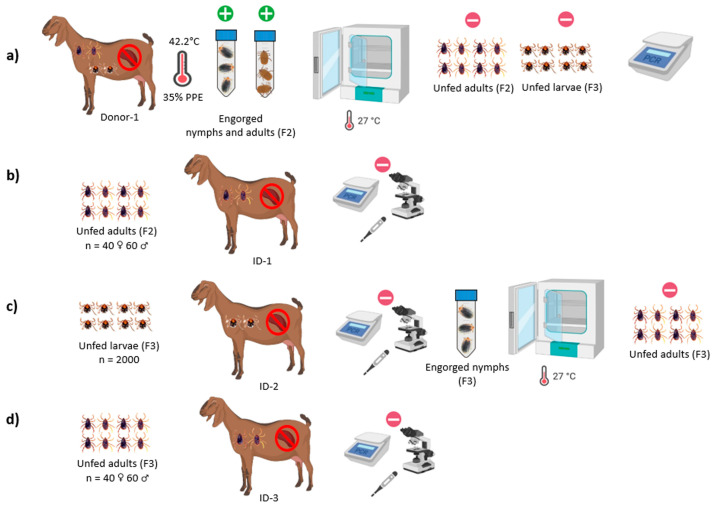
(**a**) Blood feeding of immature and adult stages of *R. bursa* on the donor goat infected with *B. aktasi*. (**b**) Infestation of #ID-1 with unfed adult *R. bursa* (F2) obtained from larvae infested with #Donor-1 (transstadial transmission). (**c**) Infestation of #ID-2 with unfed *R. bursa* larvae (F3) obtained from females infested with #Donor-1 (transovarial transmission). (**d**) Infestation of #ID-3 with unfed adult *R. bursa* (F3) obtained from larvae (F3) infested with #ID-2 (transovarial transmission). Figure 2 was created with BioRender.com (https://www.biorender.com/ accessed on 12 June 2024). +, −; represent nested PCR positive and negative, respectively.

Two experiments were performed in this study to determine the vector competence of *R. bursa* for the transmission of *B. aktasi*. Our recent experimental study demonstrated that *B. aktasi* induced high parasitemia near to 41% in immune-suppressed indigenous goats [25]. Therefore, immune-suppressed local goats were used in these experiments to ensure that high numbers of parasites ingested by the ticks. Representative schema of the experimental studies are presented in Figure 1 and Figure 2.

#### 2.4.1. Experimental Infection of the Donor Goat

An immune-suppressed indigenous goat was used in this study as a donor to facilitate the acquisition of *B. aktasi* by both immature (larva, nymph) and adult *R. bursa* during blood-feeding. For experimental infection, 15 mL of the thawed cryopreserved stabilate of *B. aktasi* with 10% parasitemia was administered to the splenectomized donor goat by intravenous injection (Figure 1b). Following parasite inoculation, 4 consecutive doses of intramuscular dexamethasone (Vetakort^®^ 4 mg, Vetas, Istanbul, Türkiye; 20 mg daily dose) were also administered to the donor [11,25,28]. The donor was monitored daily for clinical and parasitological findings of babesiosis. Blood samples with EDTA collected from the donor were utilized for hematological analysis and DNA extraction.

#### 2.4.2. Acquisition of *B. aktasi* by *R. bursa* in the Clinically Infected Donor Goat

Before the inoculation of the parasite, two feeding capsules made from EVA foam as described by Almazan et al. [29] were attached to the back of the shaved thorax area of the donor goat. Two thousand unfed larvae and a total of 25 unfed adult ticks consisting of 10 females and 15 males (F2) derived from the laboratory colony were applied to the separate feeding capsules (Figure 1b). The larvae were applied 3 days before parasite inoculation, while the adult ticks were applied 6 days after incubation and allowed to feed on the donor goat until they were engorged. Following detachment of ticks, all the engorged nymphs and adult ticks were harvested in separate ventilated sealed plastic tubes as previously described [30] (Figure 2a).

The representative 20 individual engorged nymphs (F2) were stored in a deep freezer (−20 °C) until use for DNA extraction and then PCR analysis for the presence of *B. aktasi*. The remaining engorged nymphs were kept in an incubator at 25 ± 1 °C, 70 ± 10% RH, and allowed to mount into unfed adult ticks. They were monitored daily, and their progression to the adult stage was recorded. Once molted, the unfed adult ticks were sorted by sex, and the total number of females and males was documented. After the engorged nymphs molted to unfed adult ticks, a total of 25 F2 unfed adult pools containing 3 ticks in each pool were stored in the deep freezer for DNA isolation and PCR analysis. The remaining unfed adult ticks assumed to be infected with *B. aktasi* were kept at room temperature until use for the infestation of #ID-1 recipient goat (transstadial transmission).

All the engorged female ticks (n = 10) were also kept in the incubator and allowed to lay eggs. After the completion of oviposition, the engorged female carcasses were removed from the incubator and cut in half with a sterile scalpel and placed in Eppendorf tubes for DNA extraction. When changes in the color of the egg mass were observed, 5 separate egg pools containing 150 eggs in each pool were created from the egg mass laid by each of 10 female ticks. Thus, a total of 50 egg pools (5 pools for each of 10 engorged female ticks) were maintained in the incubator until new progeny larvae hatched from the eggs, and then these larvae pools (F3) were also used for DNA isolation and PCR detection. In addition, 200 eggs were collected from each of the egg masses belonging to the 10 female ticks, resulting in a new cluster of egg masses containing 2000 eggs. The new cluster of egg masses was maintained in the incubator for the hatching of the F3 larvae (Figure 2a).

#### 2.4.3. Transstadial and Transovarial Transmission Experiments of *B. aktasi* to the Recipient Goats by *R. bursa*

In these experiments, three splenectomized recipient goats (#ID-1, #ID-2, and #ID-3) free of *Babesia*, *Theileria*, and *Anaplasma* were used. #ID-1 recipient goat was infested with a total of 100 F2 unfed adult ticks (40 females and 60 males) derived from the engorged nymphs that completed their immature stages by feeding on #Donor-1 (transstadial transmission) (Figure 2a,b). Certain *Babesia* species, such as *B. bigemina* and *B. ovis*, are not transmitted by vector ticks to vertebrate hosts during their larval and nymphal stages, respectively [1,15]. Therefore, in this study, both immature and adult *R. bursa* were used to infest #ID-2 and #ID-3 recipient goats for the transovarial transmission, respectively. Specifically, 2000 F3 larvae obtained from the engorged females that had fed on #Donor-1 were used to infest #ID-2 recipient goat. Subsequently, #ID-3 was infested with unfed adult *R. bursa* derived from the engorged nymphs collected from #ID-2 recipient goat (Figure 2c,d). All the recipient goats (#ID-1, #ID-2, and #ID-3), infested with unfed immature and adult *R. bursa*, were injected with 4 consecutive doses of intramuscular dexamethasone (Vetakort^®^ 4 mg, Vetas, Istanbul, Türkiye; 20 mg daily dose) following tick infestation. To monitor parasitemia and *B. aktasi* detection in tick-infested animals (#ID-1, #ID-2, and #ID-3), peripheral blood samples were collected daily from the ear tip for microscopic examination, and EDTA blood samples were collected from the jugular vein for molecular analysis until the 40th day post-experimental infection.

The recipient goats were monitored for clinical symptoms of babesiosis (rectal temperature, anemia, jaundice, hemoglobinuria, and appetite) and the presence of *Babesia* spp. (microscopy and nPCR) from the 5th to the 40th day after tick infestation.

### 2.5. DNA Isolation from Tick and Blood Samples and nPCR

The tick samples used in this study are described in Table 1. Briefly, 20 individual engorged nymphs (F2), 10 engorged female carcasses (F2), 25 unfed adult pools (F2) from #Donor-1, 50 unfed larval pools (F3), and 25 unfed adult pools (F3) from #ID-2 were collected in Eppendorf tubes. They were crushed in liquid nitrogen using a micro pestle [31].

Genomic DNA was isolated from tick and blood samples using a PureLink^TM^ Genomic DNA Mini Kit (Invitrogen Corporation, Carlsbad, CA, USA) as described by the manufacturer’s procedure. *Babesia*-free *R. bursa* ticks from the laboratory colony were used as a negative control to avoid cross-contamination during the extraction. Nested PCR was performed to test for *Babesia*/*Theileria* spp. and *Anaplasma* spp. using two sets of genus specific primers, Nbab1F-Nbab1R/RLBF2-RLBR2 [32,33] and EC9-EC12A/16S8FE-B-GA1B [34,35], respectively. Positive and negative controls were utilized in each PCR reaction. The PCR reaction and thermal cycling conditions were as described by Ozubek et al. [25]. Ten microliters of the PCR products were run on 1.4% agarose gel for 30 min and monitored on the Quantum Vilber Lourmat (Marne-la-Vallee, France) gel imaging system. In addition, a PCR was also performed using 16S  +  1 and 16S − 1 primers to check the DNA of *R. bursa* as previously described [36].

The *Babesia* spp. detected in tick and blood samples through PCR analysis was confirmed as *B. aktasi* by DNA sequencing. For this, a region of approximately 1600 bp of the *18S rRNA* gene was amplified using the primers Nbab1F and Nbab1R [32]. The resulting nucleotide sequences were then compared with those in the NCBI database using BLAST analysis.

### 2.6. Microscopic Detection

Thin blood smears were fixed with methanol and stained with 10% giemsa. The stained smears were examined for the presence of intra-erythrocytic stages of *Babesia* spp., *Theileria* spp., and *Anaplasma* spp. under a light microscope (×1000 magnification). The percentage of parasitized erythrocytes (PPE) was determined by analyzing a minimum of 20 microscopic fields, as outlined in the methodology of the previous studies [25,27].

## 3. Results

### 3.1. Experimental Infection of B. aktasi in Donor Goat

**Figure 3:**
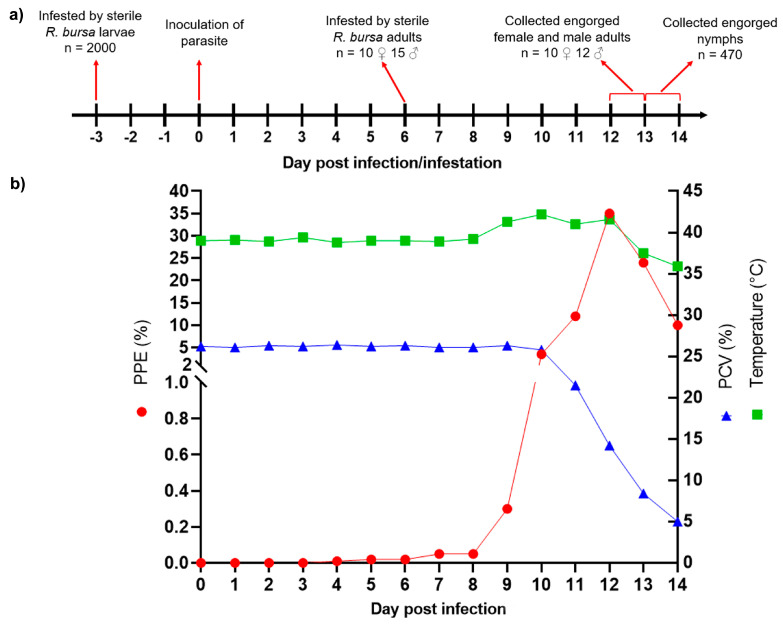
Tick infestation process and course of clinical infection in donor goat. (**a**) Tick infestation and infection timeline of #Donor-1 for tick acquisition and transmission experiment. (**b**) Course of parasitemia (PPE), rectal temperature (°C) and PCV (%) changes during infection in the donor goat (donor was euthanized on the 14th day post-infection due to severe clinical babesiosis).

Following the injection of *B. aktasi* stabilate, severe clinical infection with fever, anemia, jaundice, hemoglobinuria, loss of appetite, and rapid breathing occurred in the goat. On the fourth day after parasite inoculation, the first piroplasms were observed in the peripheral blood smears. Correlated with the appearance of intracellular parasites, an increase in rectal temperature up to 42.2 °C was measured. Parasitemia gradually increased, and the peak parasitemia was determined as 35% on the 12th day post-parasite inoculation, then declining to <10% over the following days. As the parasitemia increased, a decrease in the packed cell volume (PCV) value was observed, and the decrease in the PCV value reached 5% on the 14th day post-infection. The goat could not recover from severe babesiosis and was humanely euthanized on the 14th day after infection, following the collection of all engorged nymphs (Figure 3b). The clinical infection in the donor goat was confirmed to be caused by *B. aktasi* through PCR followed by DNA sequencing.

### 3.2. Outcome of Acquisition of B. aktasi by Immature and Adult Stages of R. bursa Fed on the Clinically Infected Donor Goat

The attached larvae, having imbibed parasite-containing blood from the infected donor goat, completed their feeding and molting on the host. These larvae were collected as engorged nymphs from #Donor-1 between the 13th and 14th days post-infestation (Figure 3a). A total of 490 engorged nymphs were collected from #Donor-1, and 20 engorged nymphs were individually examined for the presence of *B. aktasi* using nPCR. Representative sequencing analysis confirmed the presence of *B. aktasi* DNA in all the engorged nymphs. The remaining 470 engorged nymphs were incubated under the conditions specified in Section 2.4.2 to obtain unfed adult ticks, resulting in 387 (181 females, 206 males) unfed adult *R. bursa*. Of these 387 unfed adult *R. bursa*, the representative 75 unfed adult ticks divided into 25 pools containing 3 ticks in each pool were screened by the PCR for the presence of *Babesia* spp. However, the parasites’ DNA was not detected in any of the pools (Table 2). These findings indicated that the immature stages of *R. bursa* ingested *B. aktasi* from the infected donor goat during the feeding period, but the infection could not be transferred to the next stage, unfed adult ticks, via transstadial transmission.

Of the 25 adult *R. bursa* (10 females and 15 males) placed on the #Donor-1, all female and 12 male ticks that survived through feeding were collected from the host as engorged ticks between the sixth and seventh days post-infestation (Figure 3a). After being placed in the incubator, the engorged female ticks began laying eggs from the fourth day onwards. The hatching of larvae from the eggs started between days 28 and 34, and lasted for 6–13 days (F3). After completion of oviposition, all engorged female carcasses screened by nPCR were positive for the evidence of *Babesia* spp.; however, there was no amplification of the parasite DNA in any of the 50 larval pools emerging from each of the engorged female ticks (Table 2). These results indicate that *R. bursa* acquired *B. aktasi* while feeding on the infected goat during their adult stage, but they were unable to transmit the parasite to new progeny larvae (F3) via transovarial transmission. Furthermore, molecular and microscopic findings related to *Anaplasma* and *Theileria* spp. were negative in the larvae, nymphs, and adult ticks.

### 3.3. Babesia Aktasi Is Not Transstadially and Transovarially Transmitted to Immune-Suppressed Goats by R. bursa

Although molecular data obtained from ticks indicated that *R. bursa* does not possess vector competence for *B. aktasi*, the transstadial and transovarial transmission experiments were performed on the three immune-suppressed recipient goats to confirm these findings. After the immature and adult tick infestations, the development of clinical signs and the appearance of piroplasms in peripheral blood smears were expected in the recipient goats. However, no clinical or parasitological findings were encountered in the recipient goats for 40 days post-infestation. Moreover, no nPCR-positive products for *B. aktasi* were obtained from the recipient goats’ DNA samples during this period. Thus, the in vivo transmission feeding experiments confirmed the molecular data obtained in ticks and indicated that *R. bursa* was not a competent vector for the transmission of *B. aktasi*.

## 4. Discussion

Babesiosis was the first vector-borne vertebrate disease discovered and has been shown to be spread by ixodid ticks [37]. The terms “vectorial capacity” and “vector competence” are commonly used to describe the ability of an arthropod to serve as a vector for a disease agent [38]. Vectorial capacity is influenced by vector density and environmental determinants, while vector competence is defined as a component of vectorial capacity influenced by genetic factors affecting a vector’s ability to transmit a pathogen [39]. *Babesia* species belonging to the *Babesia* s.s. clade (clade VI) are transmitted by ticks both transstadially and transovarially [2]. Although both transmission routes play a role in the spread of the disease, transovarial transmission from the female tick to new larval ticks is crucial for the enzootic cycle of babesiosis [8]. Many ixodid ticks have been reported as potential vectors for *Babesia* species [1,10]. Most of these reports are based on the correlation between the presence of ticks and the occurrence of the disease, rather than objective experimental evidence of transmission. The current approach, which relies primarily on the correlation between the presence of ticks and the occurrence of the disease, may obscure the true epidemiology of the disease and result in the misidentification of *Babesia* species [1,8]. It has been suggested that, for a tick’s vector competence to be accepted, the existing epidemiological relationship should be supported by tick transmission experiments [9]. *Babesia aktasi* is a newly discovered agent with a wide distribution in indigenous goats in the Mediterranean region of Türkiye [23,24]. Our recent molecular field survey revealed that *R. bursa* may be a potential vector of the parasite due to the overlap of high prevalence of infection in goats with dominant infestations by *R. bursa* [24]. However, this correlation needs to be supported by experimental transmission data. Therefore, it is necessary to elucidate the transmission ability of *R. bursa* for *B. aktasi*. Identifying the vector tick for the newly described *B. aktasi* is crucial for understanding the biology of the parasite and for developing and implementing appropriate control measures to prevent caprine babesiosis.

To determine whether an ixodid tick has vector competence for any *Babesia* s.s., the most established experimental approach involves three distinct steps. These include the ingestion of piroplasms by laboratory-reared ticks feeding on a host infected with *Babesia* spp., the transfer of the acquired parasites to the next developmental stage (transstadial transmission) or next generations of larval ticks (transovarial transmission), and finally, the transmission of the parasite to a susceptible host [40]. Unless these three processes are experimentally demonstrated, it may not be claimed that a tick species has vector competence [40,41]. Thus, here, we elucidated the ability of laboratory-reared *R. bursa* to maintain *B. aktasi* infection from immature ticks to the next stage, unfed adult ticks via transstadial, and female ticks to new-generation larvae via transovarial transmissions.

In this study, experimental infection of a donor goat with *B. aktasi* stabilate resulted in 35% parasitemia on the 12th day post-parasite inoculation accompanied by high fever up to 42.2 °C. Severe clinical symptoms (anemia, jaundice, hemoglobinuria, loss of appetite, and rapid breathing) occurred in the goat, similar to our previous experimental study using the same isolate [25]. The transstadial feeding experiment revealed that the majority of unfed larvae (F2) placed on the infected donor goat were detached from the host as engorged nymphs. Representative DNA samples from these engorged nymphs screened by nPCR were positive for evidence of *Babesia* spp. DNA. However, none of the DNA samples from the unfed adult pools emerging from the engorged nymphs showed positive amplification for the parasite DNA. These results indicated that *R. bursa* was unlikely to contribute to the transstadial transmission of *B. aktasi*. This finding is consistent with those of previous studies documenting the absence of *Anaplasma phagocytophilum* and *Borrelia burgdorferi* s.s. DNA detection in molting to the next stage of *Haemaphysalis longicornis* collected from experimentally infected goats and mice, respectively [42,43]. Our finding is also in agreement with studies indicating that *Hae. longicornis* and *Hae. concinna* could acquire *B. burgdorferi* sensu lato and *B. garinii* while feeding on infected mouse hosts in the immature stages, but the infection was lost during the molt to the subsequent life stage [44,45]. It has been reported that there may be negative interactions among ovine *Theileria*, *Babesia*, and *Anaplasma* species in co-infected sheep [46]. Therefore, #Donor-1 and ticks derived from it were also screened for the presence of these pathogens.

Similar to the transstadial experiment, our transovarial feeding experiment showed that the engorged female carcasses screened by nPCR were positive for the evidence of *B. aktasi* DNA, but no PCR positivity was detected in any of the larval pools emerging from each of the engorged female ticks. This result demonstrated that *R. bursa* acquired the piroplasms of *B. aktasi* while feeding on the infected donor goat during their adult stage, but they were unable to transmit the parasite to new progeny larval ticks via transovarial transmission. This finding corresponds with the previous results indicating that *R. sanguineus and Hy. marginatum* could not transovarially transmit *B. ovis* to the splenectomized sheep [47].

In this study, molecular findings obtained from ticks provided strong evidence that *R. bursa* did not have vector competence for the transmission of *B. aktasi*. Demonstrating that the pathogen does not amplify in DNA samples from individuals that completed their development by feeding on an infected host and molting to the next stage or the new generation is a significant indicator for vector competence [12,48]. However, Bonnet and Nadal [9] suggested that ticks presumed to be infected should be allowed to feed to repletion on a susceptible host, and then the host should be tested for the pathogen using clinical, parasitological, and molecular methods. In an experimental study conducted in China [12], *Hae. qinghaiensis* and *Hae. longicornis* ticks infected with *Babesia* sp. BQ1 (Lintan) and placed on sheep showed no clinical signs or microscopic findings in the recipient sheep. However, genomic DNA of the parasite was detected in the sheep from the 7th to the 32nd days after tick infestation. This result was interpreted to mean that *Hae. qinghaiensis* and *Hae. longicornis* transmitted *Babesia* sp. BQ1 (Lintan) to the sheep, suggesting that both ticks might be potential vectors of *Babesia* sp. BQ1 (Lintan) [12]. In contrast to the study conducted in China, no clinical signs and parasitological findings were detected in the immune-suppressed recipient goats in the present study. Moreover, no PCR evidence of *B. aktasi* was detected in DNA samples from recipient goats monitored for 40 days post-infestation. Our results are consistent with the previous study conducted on mice by Kusakisako et al. [49], which revealed that *Hae. longicornis* nymphs emerging from larvae fed on mice infected with *B. microti* did not transmit the parasite to *Babesia*-free splenectomized mice.

## 5. Conclusions

In conclusion, the findings obtained in this experimental study revealed that immature and adult stages of *R. bursa* acquired *B. aktasi* while feeding on the clinically infected donor goat during their feeding period. However, this tick was unable to transmit the parasite to the next stage or new progeny larvae via transstadial or transovarial transmissions, respectively. In addition to *R. bursa*, other tick species, such as *R. turanicus* s.l., *R. sanguineus* s.l., *Dermacentor raskemensis*, and *Hy. Marginatum*, are present in the study area and could potentially act as vectors for *B. aktasi* [50]. These species’ overlapping activity periods and ecological niches could influence the transmission dynamics of *B. aktasi*. Further tick transmission studies are needed to identify and investigate tick vectors involved in the transmission of *B. aktasi* and to develop targeted control strategies to mitigate the impact of the parasite on goat populations.

## Figures and Tables

**Table 1:** Different developmental stages of *R. bursa* used for DNA extraction and nPCR analysis.

Tick Stage (Generation)	Source of Ticks	No. of Ticks in Each Pool	No. of Pools	No. of Total Ticks
Engorged nymph (F2)	#Donor-1	1 *	20	20
Engorged Female carcasses (F2)	#Donor-1	1 *	10	10
Unfed adult tick (F2)	#Donor-1	3	25	75
Unfed larvae (F3)	#Donor-1	150	50	7500
Unfed adult ticks (F3)	#ID-2	3	25	75

* Individual tick sample.

**Table 2:** Results of nPCR analysis for the efficiency of acquisition and transstadial and transovarial maintenance of *B. aktasi* in *R. bursa* ticks fed on the infected donor goats.

Tick Stage and Feeding Status	Generation	Transmission Route	No. PCR-Positive Ticks/No. Ticks Tested (% Infection)
Engorged nymphs (individual)	F2	Transstadial	20/20 (100%)
Unfed adults (pool)	F2	Transstadial	0/25
Engorged female carcasses	F2	-	10/10 (100%)
Unfed larvae (pool)	F3	Transovarial	0/50
Unfed adults (pool)	F3	Transovarial	0/25

## Data Availability

The original contributions presented in the study are included in the article, further inquiries can be directed to the corresponding author.
